# Climate Change, Community Action, and Health in the Anglophone Caribbean: A Scoping Review

**DOI:** 10.3389/phrs.2023.1605843

**Published:** 2024-01-12

**Authors:** Sonja Lynn Myhre, Michelle Scobie, Eija Meriläinen, Ilan Kelman, Unni Gopinathan

**Affiliations:** ^1^ Norwegian Institute of Public Health (NIPH), Oslo, Norway; ^2^ Institute of International Relations, The University of the West Indies St. Augustine, St. Augustine, Trinidad and Tobago; ^3^ School of Humanities, Education and Social Sciences, Örebro University, Örebro, Sweden; ^4^ Hanken School of Economics, Helsinki, Finland; ^5^ Institute for Risk and Disaster Reduction and Institute for Global Health, University College London, London, United Kingdom; ^6^ Department of Global Development and Planning, University of Agder, Kristiansand, Norway

**Keywords:** wellbeing, climate change, health, community action, Caribbean

## Abstract

**Objective:** This scoping review investigates the status of research focusing on the nexus of community action, climate change, and health and wellbeing in anglophone Caribbean Small Island Developing States (SIDS).

**Methods:** This review was guided by Arksey and O’Malley framework and utilized the PRISMA-ScR checklist. We searched Medline/OVID, PsychInfo, VHL, Sociological Abstracts, Google Scholar, and Scopus to capture interdisciplinary studies published from 1946 to 2021.

**Results:** The search yielded 3,828 records of which fourteen studies met the eligibility criteria. The analysis assessed study aim, geographic focus, community stakeholders, community action, climate perspective, health impact, as well as dimensions including resources/assets, education/information, organization and governance, innovation and flexibility, and efficacy and agency. Nearly all studies were case studies using mixed method approaches involving qualitative and quantitative data. Community groups organized around focal areas related to fishing, farming, food security, conservation, and the environment.

**Conclusion:** Despite the bearing these areas have on public health, few studies explicitly examine direct links between health and climate change. Research dedicated to the nexus of community action, climate change, and health in the anglophone Caribbean warrants further study.

## Introduction

*The Intergovernmental Panel on Climate Change (IPCC) Sixth Assessment report* warns that Small Island Developing States (SIDS) are increasingly impacted by sea level rise, heat waves, increasing frequency and intensity of hurricanes, storm surges, increase in rainfall, and volatility of precipitation patterns [1]. In turn, these events often lead to erosion, landslides and flooding, droughts, destruction of coral reefs, degradation of coastal environments, contamination of water supplies and diminishing availability of freshwater, natural habitat destruction, and increasing invasive species—all with serious implications for livelihoods, productivity, health, food and water security, and ecosystems throughout the Caribbean [2, 3].

In 2017, the World Health Organization launched the Small Island Developing States Initiative on climate change and health recognizing that “small islands are fragile ecosystems populated by resilient people who have been able to cope with environmental threats over centuries, and for some, over millennia” (pg. 10) [4]. At the regional level, *The State of the Climate in Latin America and the Caribbean 2021 report* by the World Meteorological Organization indicates that continued warming trends, droughts, heatwaves, tropical cyclones and floods are responsible for “the loss of hundreds of lives, severe damages to crop production and infrastructure and human displacement” [5]. With even greater granularity, the *State of the Caribbean Climate* report (2022), authored by thirty-one authors from three Caribbean institutions, provides a rich repository of Caribbean climate data documenting climate variability, future projections, and impacts on climate sensitive sectors [6].

Our focus on community action research in the Caribbean emanates from the susceptibility of these communities to climate change [7–12]. Given that government, private sector, and supranational responses may be slow and/or insufficient to respond to climate-related impacts, increasingly, attention has shifted towards volunteer community-based organizations and other informal groups that may contribute to addressing community priorities and needs [13]. While national and regional engagement is essential (i.e., building codes, planning regulations, taxes, and financial incentives), Betzold observed that, “[t]he scholarly consensus is clear: adaptation is fundamentally a local issue, and local involvement, participation and ownership is a central precondition for successful adaptation” [14].

Although climate change is a global problem necessitating global, national, and regional solutions, many impacts of climate change are experienced at the community level and communities respond and act in response to these impacts [13]. Local knowledge, traditions, and values, for example, need to be considered underscoring the importance of involving communities in participatory processes [15]. Along these same lines, Kelman noted the value of listening to and integrating local voices from SIDS in climate change processes that embrace local perspectives, priorities, and needs [16, 17].

**FIGURE 1:**
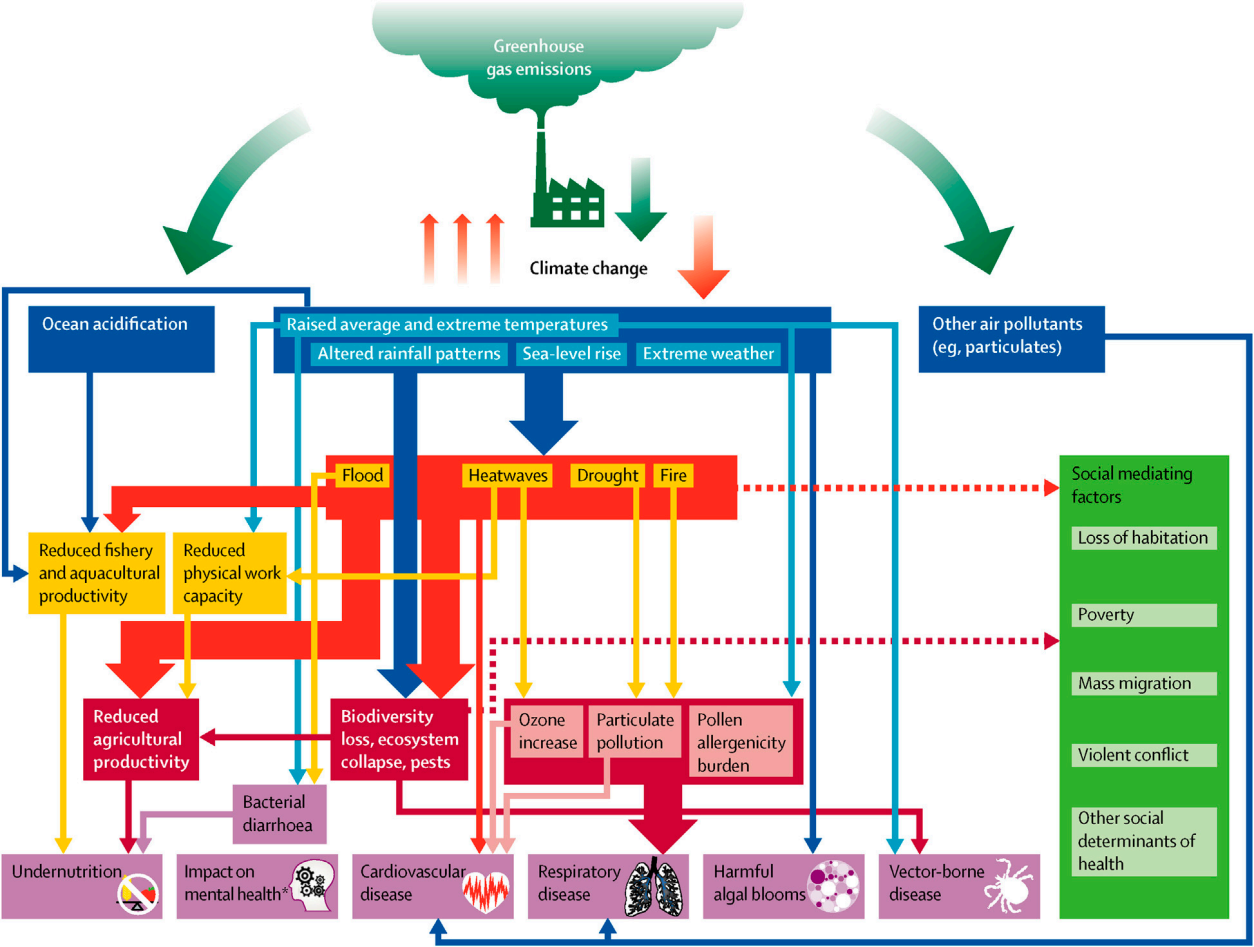
Climate change and health pathways (scoping review, global, 2021–2022) (reproduced with permission from [3]).

Recent Lancet Countdown on Climate Change and Health reports provide exhaustive documentation of the health impacts from climate change including injuries, undernutrition, infectious, respiratory, and cardiovascular disease, allergies, poisoning and mental illness [2, 3]. Figure 1 illustrates several potential pathways between climate change and health such as compromised water resources, biodiversity loss, and increased food insecurity that may disrupt livelihoods sensitive to climate (i.e., farming, fishing, and ecotourism). In contexts where communities already suffer negative effects of global financial shocks, geographical marginalization, and poverty, this may compound economic hardship and, in some cases, contribute to food insecurity and mental distress.

**FIGURE 2:**
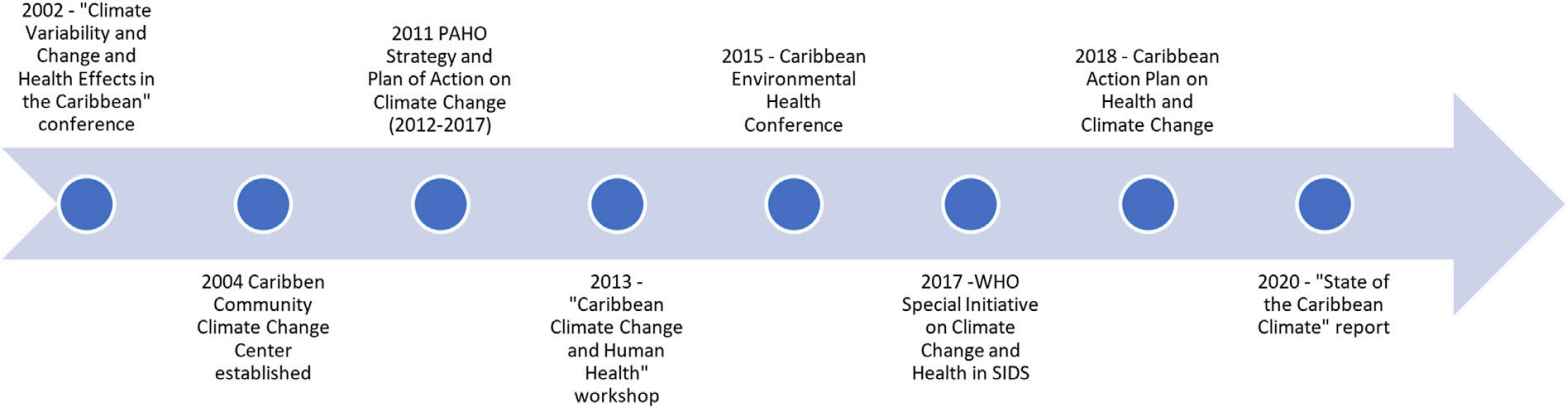
Timeline of climate change and health work in the Caribbean Small Island Developing States (scoping review, Caribbean, 2021–2022).

The longstanding interest by researchers in this field is evident, exemplified by the Caribbean hosting a conference in 2002 dedicated to climate change and health. During this same timeframe, a climate database was established and local research on dengue outbreaks commenced [18]*.* Later, the PAHO Strategy and Plan of Action (2011), Caribbean-based workshops (2013), conferences (2015, 2018), and reports (2017, 2020) focusing on climate change and health underscore the importance of this topic in the region. Figure 2 provides an overview of historic milestones reflecting climate change and health research activities in the Caribbean.

Our research builds on this long history as well as on previous reviews that have focused broadly on climate change and health in SIDS or the Caribbean [13, 19, 20]. The aim of this research is to analyze the status of research on the nexus of climate change, health, and community action in the anglophone Caribbean to provide insights on how community action in the Caribbean is addressing climate change and health challenges. This review aims to answer the following research questions:

• How do community-based stakeholders respond to climate change’s direct and indirect health impacts in anglophone Caribbean countries?
• What are the key barriers and facilitators community-based stakeholders face in addressing health impacts of climate change?
• How is climate change, health and livelihoods interlinked in anglophone Caribbean countries?

## Methods

### Study Design

This study was designed using a scoping review approach given that the intersection of climate change, health and community action represents an emerging area of research with undefined disciplinary boundaries. Our broadly formulated research questions require mapping relevant literature that was expected to reside in various disciplines using several interpretive approaches as our aim was to identify the nature and extent of research evidence dedicated to this topic [21–23].

We used the Preferred Reporting Items for Systematic Reviews and Meta-Analyses (PRISMA) checklist to guide our study. We relied on Arksey and O’Malley’s five-stage framework to inform and guide our process [23]. These stages comprise developing research questions, identifying relevant studies, study selection, synthesizing and interpreting key pieces of information (i.e., “charting the data”) and collating, summarizing, and reporting the results. The scope and study terms are described in the Box 1.

**Table:** 

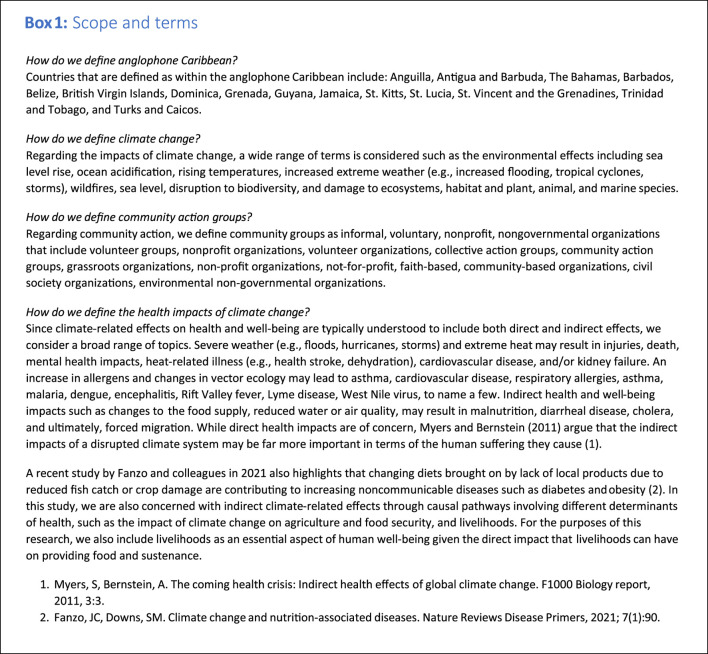

### Search Strategy and Selection Criteria

We systematically searched several databases including Medline/OVID, PsychInfo, VHL, Sociological Abstracts, Google Scholar, and Scopus to capture a broad range of multi- and interdisciplinary studies. Our exclusion criteria limited publications to English and peer-reviewed journal articles and excluded conference or workshop proceedings, abstracts, and dissertations. The inclusion criteria stipulated that articles focus on all four topical areas: geographic area within the anglophone Caribbean, community-based stakeholders, groups, or organizations, climate change and health and wellbeing.

The timeframe includes articles published between 1999 until July 2021. The search strategy was designed and conducted by an information specialist at the Norwegian Institute of Public Health. The full search strategies for each database are available in Supplementary Appendix SA1 (Supplementary Material). Two independent researchers screened records by title and abstracts using Covidence. Disagreements were resolved by discussions among the two researchers that led to a conclusion. Full text screening was completed independently by two researchers resulting in the identification of the final set of articles. This search strategy was supplemented by a consultation with co-authors as well as a review of citations using Web of Science that identified three additional records.

In terms of researcher reflexivity, our team was multidisciplinary which was important since we collectively needed to be familiar with literature originating from different disciplines. Moreover, one of the main researchers is from the Caribbean which added critical insight to the review process. Several pilot searches were conducted to assess the relevance of the retrieved articles and to optimize the search strategy. How articles are indexed can differ among databases, especially social science and biomedical databases. To minimize the risk of overlooking relevant articles, authors familiar with the literature from this field and region compiled tracer articles to enhance the robustness of the search.

### Data Extraction

Two researchers independently extracted data from each article into a predefined Excel format. A third researcher compared the two data extraction files and documented areas of agreement and dissimilar findings. The two researchers then resolved discrepancies in the data extraction file. Data extracted from each article included information pertaining to first author’s institutional affiliation, study design, country/setting, methods (qualitative, quantitative, mixed methods, case study), analytic, theoretical, or conceptual framework, type of community organization, intervention, outcomes, focal topic of article, climate change focus, indirect or direct health impact, and government and community interaction.

### Analytic Framework

After reviewing frameworks on climate change, community action, and coastal communities [24, 25], we opted to modify the local adaptive capacity framework developed by Jones’ framework with the primary difference reflecting the category of agency that Jones and others refer to as a cross-cutting issue [24, 25]. We used this framework as a guiding tool to assist in structuring our analysis. The five dimensions of adaptive capacity that we explore include: resources/assets, education/information, organization/governance, innovation/flexibility, and agency/efficacy. These categories are broad in scope, interdependent and often have overlap. While we acknowledge that many different frameworks, models, and perspectives offer other viable ways to analyze content, we suggest that a modified framework offers a useful lens to understand factors that influence, interact, enable and/or constrain local level adaptive capacity. Table 1 provides descriptions of these dimensions with examples from the literature.

**TABLE 1:** Dimensions of adaptive capacity, description, and examples from the literature (scoping review, Caribbean, 2021–2022).

Dimensions	Description	Examples
Resources/assets	Financial, materials, equipment, property, infrastructure, or services that facilitate adaptation	Assets such as livestock, land, and home ownership [36]
		Fishing resources, materials, and equipment [29, 35]
Education/information	Information, education, training, and skills that create competencies and capacities contributing to adaptation	Knowledge systems, knowledge cultures, and learning processes [31]
		School curriculum, formal and informal educational community programs [30]
Organization/governance	Organization and coordination approaches (i.e., governance) supporting collective action	Engagement of community with research institutions, media, and village council [33]
		Partnerships and collaborative cooperation with NGOs [11]
Innovation/flexibility	Unique and flexible strategies designed to address adaptation problems	Livelihood diversification [28]
		Fishing strategy diversification [27, 35]
Efficacy/Agency	Self-efficacy, belief in ability to achieve and achieve results	Self-efficacy [34]
		Fisherfolk tradition/ethic [35]

## Results

### Studies and Study Characteristics

**FIGURE 3:**
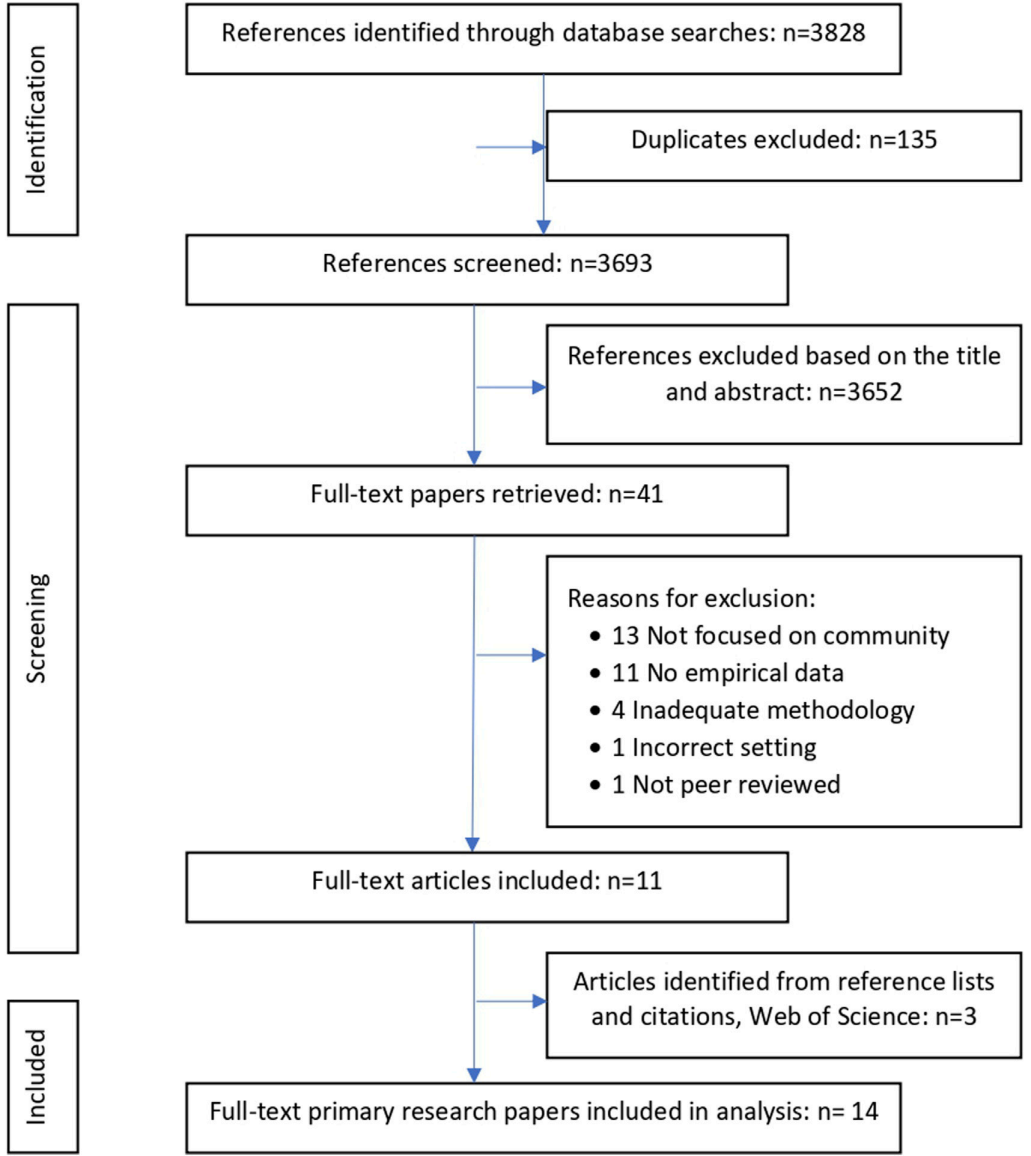
PRISMA (preferred reporting items for systematic review and meta-analyses) flowchart (scoping review, Caribbean, 2021–2022).

We identified 3,828 potential records (excluding duplicates) for title and abstract review. This screening process led to the full-text screening of forty-one records of which eleven were deemed suitable for full data extraction. A review of the citations list of each of these eleven articles identified additional relevant articles bringing the total to fourteen (See Figure 3: PRISMA).

Table 2 provides details on the articles and study characteristics. Date of publication ranges from 1999 to 2020 with the preponderance of articles published after 2015. These articles focused on the following countries in the anglophone Caribbean: Anguilla, the Bahamas, Belize, Dominica, Grenada, Jamaica, St. Vincent and the Grenadines, Trinidad and Tobago, and Turks and Caicos. The majority of first authors had affiliations in high income countries with one from the Caribbean: United Kingdom [6], Germany [2], Ireland [2], Norway [2], Canada [1], and Jamaica [1]. In terms of the focal group of interest, the studies focused on fishing community groups, farming and agricultural groups, conservation and environmental groups, forestry management groups, tourism operators, water management, disaster risk management organizations, and civil society stakeholders.

**TABLE 2:** Study characteristics (scoping review, Caribbean, 2021–2022).

#	First author, year	First author’s country affiliation	Country	Participants/stakeholders	Climate change focus	Health or wellbeing focus	Methods/Analytic framework	Study aim
1	Lundy, 1999 [37]	Ireland	Jamaica	General public, environmental NGOs, international stakeholders	Conservation of the environment	Indirect impact on health from environmental degradation	Interviews with eNGOS and international stakeholders	Investigates community participation in conservation efforts
2	Tompkins, 2004 [34]	United Kingdom	Trinidad and Tobago	Multi-community stakeholders	Hurricanes, flooding	Increasing pathogens, diseases, and pests	Theory of social learning and co-management by governments and resources	Explores how co-management of resources may expand networks and increase social resilience
3	Suarez, 2008 [32]	United Kingdom	Bahamas	Red Cross and poor community members	Hurricanes, flooding	Preparedness, safety, disaster survival	Case study investigating the use of community participation of video production	Community level adaptation using video participatory approach
4	Forster, 2014 [35]	United Kingdom	Anguilla	Fishers and marine-based tourism operators	Hurricanes, impacts on fish stocks, sea level rise	Indirect impact of climate change on marine dependent livelihoods (i.e., fishers and marine-based tourist operators)	Case study focusing on marine resource-dependent livelihoods and their response(s) to hurricanes and future environmental change impact on resource and livelihood security	Investigates climate change impacts on marine systems to explore resilience of marine-dependent livelihoods (fishers and marine tour operators)
5	Middlebeek 2014 [9]	Netherlands	Trinidad and Tobago	Low-income urban settlement in Trinidad and Tobago	Flooding, landslides, Freshwater resources, reduction in tourism	General impact on public health	Case study of climate adaptation among St. Joseph residents and institutional actors working with climate change adaptation efforts	The study investigates the local adaptive capacity measures by residents in response to flooding and the institutional architecture that supports local climate change adaptation
6	Karlsson, 2015 [33]	Norway	Belize	Monkey River residents, focus on villagers in fishing and tourism	Coastal erosion, hurricanes	Indirect impact related to environmental impact on livelihoods such as fishing and tourism	Case study, part of a larger project examining coastal communities’ vulnerability and adaptation to multiple processes of change	Investigates how local collective action was leveraged for external support in response to severe coastal erosion that threatened the village of Monkey River
7	Baker, 2015 [29]	Wales	Turks and Caicos	Local community, Red Cross, and churches	Biodiversity loss, seagrass meadow loss	Diet, food security	Social research analysis involving interviews (n=40) conducted during three field trips; secondary data sources such as grey literature, policy documents, and official statements	Investigates ecosystem of seagrass, fisheries and food supplies and implications for food security
8	Holdschlag, 2016 [31]	Germany	Grenada and Bahamas	Civil society, environmental groups	Hurricanes, landslides, flooding	Not mentioned	Complexity theory, case studies in two settings	Disaster management and environmental governance
9	Jaja, 2016 [11]	Canada	St. Vincent and the Grenadines	Paget Farm community	Threats to freshwater availability due to saltwater intrusion, pollution, and low replenishment	Availability of freshwater for human health, plants, and animals	Case study of Paget Farm community and using social network analysis	Investigates the role of institutional integration in facilitating large scale adaptation measures that enhance community-based climate change adaptation capacity
10	Tomlinson, 2018 [36]	Jamaica	Jamaica	Farming community participating in farmer schools	Drought, flooding, hurricanes impact agricultural sector	Diet, nutrition, food security	Case study of farmer field school in Clarendon, Jamaica using interviews, semi-formal surveys, and focus groups	Investigates how farmer field schools promote pro adaptive behaviors addressing climate change
11	Petzold, 2018 [46]	Germany	Bahamas	Civil society, NGOs, and government actors in the Bahamas	Sea level rise, coastal erosion, coastal resource degradation	Ministry of Health, general impacts on health	Case study with qualitative data from island community members, NGOs, and churches	Investigates how knowledge systems impact public engagement in climate change adaptation compared to NGO involvement that may not reflect local perspective
12	Selby, 2020 [30]	Ireland	St. Vincent and the Grenadines	Community groups (SUSGREN, UIEA, CCMA, Sandwatch Beach), youth, education, and activist groups	Sea level rise, unstable weather conditions, degradation of coral reefs, dry spells, biodiversity loss, droughts, loss of fresh water	Epidemic threats	Qualitative scoping field study, case study of four different initiatives using qualitative methods such as interviews and focus groups	Study examines how different educational initiatives address climate and other related issues that also involved working with Ministries and government implemented school curriculum
13	Turner, 2020 [27]	Barbados	Dominica	Fisherfolk	Storm events, ocean warming, ocean acidification	Food security, wellbeing of fishing households	Adaptive capacity domains: assets, flexibility, social organization, learning and agency	Study investigates fishing community response to storm disruption in terms of five key adaptive capacity domains
14	Karlsson, 2020 [28]	Norway	Belize	Fisherfolk	Extreme weather events	Indirect impact of climate variability and extreme weather events on livelihoods	Case study of small-scale fishers in Belize using a framework of five domains of adaptive capacity to analyze adaptation strategies	Study examines the adaptive response strategies of small-scale fisher to extreme weather events in Belize

Most studies described their approach as a case study often using mixed methods approaches that included both qualitative and quantitative methods. Studies, for example, cited the use of semi-formal questionnaire surveys, semi-structured interviews with experts and key informants, focus groups, attendance at committee meetings, conferences, workshops, document review of annual reports, legal and policy documents, and organizational material. In some cases, ecological, meteorological, and coastal mapping data was collected.

### Climate Change Impacts

The studies address several different impacts related to climate change, including sea level rise [26], ocean warming, acidification [27], and environmental degradation (e.g., destruction of coral reefs [28], seagrass meadows [29], and mangroves [30]; an increase in non-indigenous/invasive species; changes in sedimentation processes and surface run-off; water pollution [31]; threats to ecosystems such as biodiversity loss in coastal and inland habitats [30]; increasing volatility and extremes in storms and weather patterns [9, 32, 33]; and higher temperatures [26, 34]).

Several articles discussed the impact of climate change on fishing such as more intense and frequent hurricanes that can prohibit, alter, or reduce fishing or challenges brought on by the influx of sargassum [30] and the effects on local tourism, marine biodiversity, and fishing [29, 30, 35]. Climate-related impacts on agriculture were mentioned in several articles that focused on droughts affecting crop yields, landslides impacting arable land and roads, declines in agricultural production, increased food insecurity, and diminishing freshwater availability [9, 11, 36].

### Health and Wellbeing Outcomes

A notable finding of this review is that few studies discussed *direct* health impacts from climate change. Although some studies mentioned direct impacts from extreme weather and storms in terms of safety, injury, and mental health [9, 32], articles generally discussed *indirect* health impacts related to effects on wellbeing and economic livelihoods [27, 28, 35, 36].

Studies, for example, discussed health determinants such as food insecurity [29], freshwater availability [11], the nutritional impact of ecosystem imbalance (e.g., increase in food imports, invasive lionfish species) on local communities [31], and biodiversity loss [29]. Health determinants related to livelihoods, particularly in terms of nutrition and food security, were recurrent themes in articles focusing on marine-dependent livelihoods (e.g., tourism, fishing) [29, 35] as well as the agricultural sector that can be heavily impacted by climate change [36].

### Community Action

This body of literature suggests that community action at the local level in anglophone Caribbean islands use several different approaches to address climate change and wellbeing. Community action efforts addressed several different issues including:

• Environmental education and conservation efforts (e.g., mangrove restoration, sea grass production, natural resource management) [29, 30, 37].
• Protection of marine environments (e.g., coral reefs, manage fish stocks) [28, 35].
• Agricultural development policies (e.g., staggered planting, trash barriers, mulching, contour cropping, use of cover crops, planting more heat resistant crops, companion planting) [36].
• Water conservation (rainwater harvesting, desalination projects) [9, 11].
• Nutritional impact from ecosystem imbalances (e.g., less fish, fruits, vegetables, more imports) [27, 29].
• Degradation of biodiversity (i.e., seagrass meadows) [29, 37].
• Farmer field schools and land management practices (e.g., staggered planting, mulching) [36].

A variety of strategies were utilized at the community level to address these issues. Using Noble’s classification system, these can be broadly categorized as structural/physical, social, and institutional adaptation options. Approaches falling under the category of structural/physical may include engineered and the built environment, technological and ecosystem-based responses. From our sample of included studies, building a solar-powered water desalination plant, promoting rainwater harvesting and storage [11], and restoring mangroves are examples of this type of approach [30].

Many studies described strategies that relied on educational, informational or behavioral approaches [26, 30, 31]. Selby and others, for example, described a climate change school curriculum initiative and other informal educational and informational options designed to share knowledge among students, adults and the community [30]. Farmer field schools offer another example of an educational strategy promoting agricultural practices, peer to peer learning, and sharing local agricultural practices [36]. Given the review’s dedicated focus on community action studies, fewer studies discussed institutional approaches utilizing economic, regulatory, legal, policy or programmatic options.

#### Adaptive Capacity Dimensions

As mentioned, we used a modified version of a local adaptive capacity framework to assess whether and how studies described and discussed adaptive capacity dimensions [25]. This framework provides an analytic and conceptual structure in identifying common themes, patterns, and issues.

#### Resources and Assets

Articles focusing on livelihoods, such as farming and fishing, discussed the importance of access to resources in various forms such as financial assets, physical resources (i.e., equipment), and services. Assets and resources were typically discussed in terms of human (including services), natural (e.g., sea grass meadows, coral reefs, fish stocks, etc.), financial (e.g., income, remittances cash transfers, savings, formal and informal credit), and physical capital (e.g., equipment, gear, home ownership, infrastructure, roads, irrigation). Given that several articles focused on marine and climate dependent livelihoods, the types of assets and resources discussed often related to fishing, agriculture, and tourism [27, 28, 35, 36].

Coastal livelihoods in many Caribbean countries, as described in many articles, are strongly associated with the fishing, farming and (eco)tourism industries. Regarding the fishing industry, climate-affected resources include boats, traps, fishing equipment, and storage facilities [28]. Regarding farming, assets and resources included farming equipment, livestock, irrigation supplies, water storage and rainwater harvesting facilities [36]. Tourism resources and assets related to tourist business offices and infrastructure, hotels, restaurants, as well as coral reef grounds [35]. Climate change events such as hurricanes, storms, degradation of marine resources and coral bleaching may compromise, damage, or destroy essential resources and assets, particularly among marine-dependent livelihoods thus pointing to the indirect impacts on wellbeing. Other factors, however, must also be considered such as irresponsible fishing practices leading to overfishing, damage to coral reefs from recreational snorkelers and divers, suntan lotion, which also negatively impacts marine-dependent communities.

The link between assets and resources with health relates to the notion that wellbeing is connected to livelihoods. The primary impact on assets and resources on marine-dependent and agricultural livelihoods was tied to implications related to indirect effects on wellbeing and health given that fishing and farming will impact food security, nutrition, and food safety in communities [27, 29, 35, 36].

#### Education/Information

Studies mentioned the use of training and information campaigns, farmer field schools [36], knowledge systems and knowledge cultures [31], participatory video development and competitions [32], school curriculum and environmental youth programs [30], and media campaigns and outreach [33]. Educational strategies can include formal (e.g., school curriculum, class work) or informal methods (e.g., junior ranger program designed to provide environmental education and training to students) [30].

Hallmarks of the farmer field school included hands-on training and peer-to-peer learning promoting climate adaptation practices [36]. For millennia, the global farming profession has faced challenges and has found solutions to managing weather impacts, but the farmer field schools focus on trends that could overwhelm current farming practices and provide solutions to these challenges. Moreover, the farmer field school methodology embraces locally relevant experiences, participatory approaches, peer-to-peer learning, and experiential learning strategies.

Video development and competitions offer another form of outreach and communication that aims to educate communities on climate change. Suarez and others explore how audiovisual tools can produce changes in attitudes and knowledge. They outline key elements for successful initiatives and argue that video-mediated approaches can be more successful than oral and written channels in communicating complicated scientific knowledge and especially empowering illiterate people in adaptive efforts [32].

Similarly, a study focusing on collective action and local activism by a community in response to coastal erosion mentions several informational strategies [33]. With support from external actors and resources, the community launched a collective action campaign, leveraging national media and other news outlets to build awareness and share information. These efforts were followed up with messaging delivered through television, radio, and newspaper outlets. Interestingly, research, scientific knowledge, and report findings were used to shape the outreach campaign and the media narrative which legitimized and facilitated wider dissemination. In sum, the efforts document how informational strategies were used to protect a community from flooding and coastal erosion.

#### Flexibility/Innovation

The role of flexibility and innovation in the context of adaptive capacity was discussed in several ways in the included studies. Turner and others discussed flexibility as the ability to change strategies both within and between livelihood activities [27]. Similarly, Forster discussed the idea that diversified livelihood strategies, transferable skills, and having flexible and dynamic approaches to employment may influence resilience [35]. Flexibility was also discussed in terms of fishing strategies such as selling catch directly, changing fishing tactics by switching fish species, or changing the fishing sites [35].

In a similar vein, Karlsson and Mclean’s study of fisherfolk described diversification and intensification as strategies invoked to adapt [28]. In terms of diversification, their study found that fisherfolk may engage in farming, masonry, carpentry, boating or tourism, if necessary. Tompkins and Adger discussed the importance of adaptive management practices of natural resources that contribute to strengthening community networks [34].

Innovation was mentioned by Selby and others in describing that while SIDS may be vulnerable and susceptible to climate change due to their smallness, remoteness, and localized sustainability, evidence of innovative initiatives reflected dedication and deep-rooted activism embedded in many environmental initiatives [30]. Occupational mobility and flexibility reflect the strong ties between wellbeing and agricultural and marine-dependent livelihoods. Strategies of diversification and intensification highlight strategies that are closely tied to the health and wellbeing of fishers and farmers.

#### Organization/Governance

The dimension of organization and governance was often framed around the importance of coordination and cooperation. A primary finding from Middlebeek and others’ study was that institutional communication, coordination, and partnerships among actors was essential [9]. Likewise, social network analysis conducted by Jaja and others revealed the value of vertical integration of various sectors at several scales involving different institutions [11]. Similarly, genuine involvement of local actors was the primary finding in Lundy’s study that revealed that environmental NGOs populated by local elites rather than community members can have deleterious effects on achieving policy objectives [37].

In a different vein, the study conducted by Tompkins and Adger explored the role of co-management in building community resilience by conducting a case study of a coastal community in Trinidad and Tobago that relies on coastal resources [34]. They refer to co-management as a form of collective action—the coordination of efforts among groups of individuals to achieve a common goal when individual self-interest would be inadequate to achieve the desired outcome. Finally, Baker and others’ study explores organizational and governance issues affecting seagrass meadows in Turks and Caicos using a food security lens. They conclude that weak governance structures have failed to protect marine resources resulting in implications for food security [29]. This study makes a clear link between seagrass meadow health as critical for human health given its importance to fishing grounds, seaweed cultivation, and bait collection.

#### Agency/Efficacy

Agency and efficacy were described in a few studies as having the belief or faith in the ability to accomplish or bring about change. While the studies often discussed this at the individual level, agency at the community level may reflect whether and how groups feel empowered to act. Findings from the farmer field school study, for example, noted that involvement in community-based initiatives (like the farmer field school) can influence individuals’ beliefs and risk perceptions thus reinforcing and sustaining pro-adaptive strategies [36]. In contrast, Turner and others study noted that agency has both positive and negative implications in that “strong agency can both support and undermine resilience because agency can be used to resist or oppose wider adaptation efforts” [27]. The fisherfolk ethic, for example, was described as a deep-seated desire and determination to continue fishing could be viewed as a potential deterrent to adaptation or as a reflection of dedicated agency and efficacy that could positively influence their actions thus promoting their health and wellbeing.

## Discussion

This review provides a comprehensive summary of the existing research that focuses on the intersection between climate change, community action, and health in the anglophone Caribbean [19]. The scoping review identified fourteen empirical studies focusing on nine Caribbean countries investigating local community action responses to climate change and related impacts on health. We observed a skewed distribution of researchers, with authorship being dominated by authors from high-income settings over local institutions, raising concerns about the depth of local institutional involvement in generating the knowledge. Our findings suggest that much of the research focusing on the intersection of the issues of climate change, health and community action relates to local Caribbean livelihoods such as fishing, farming, and tourism. Analysis of adaptive capacity dimensions reveals that there is often overlap between domains that may have synergistic effects.

This review underscores the need to consider a broad range of impacts when examining the influence of climate change on health. We did not limit the study inclusion criteria to research discussing direct impacts from climate change such as heat stroke or morbidity or mortality from climate change-related events. Rather, we concluded that a broad perspective that includes impacts on food, water, and the environment—that have profound effects on human health—was warranted. The rationale for this perspective is grounded in literature that has convincingly shown the crucial relationship between health outcomes and factors such as agriculture, water and food security, and environmental stressors [38–42]. This broad view enabled us to include studies of community-based responses addressing wide range of determinants affected by climate change, such as agriculture, fishing, and natural resource conservation. Interestingly, only a few included studies examined direct climate change impacts on health or health system responses [43].

Guided by the lens of the local adaptive capacity framework, the domain of education and information was a major theme for many studies. These studies demonstrate the potential for using different approaches to providing information and knowledge in forms that are accessible and useful for community members. Moreover, this review highlights the importance of recognizing that knowledge systems reflect culture, experiences, worldviews, and power relations that can result in different modes of responding to environmental pressures related to climate change [26]. Finally, innovation in climate change learning was a key theme reflecting that many initiatives in the Caribbean have utilized unique educational approaches to raise awareness, educate, and bring about behavior change which have potential value for other communities facing similar challenges [30].

Another common focus was the domain of organization and governance, often concentrating on the use of collective action approaches to solve climate change challenges and underscoring the importance of collaboration. For example, several studies focused on how communities engaged with NGOs, government actors, media, and other stakeholders to find solutions to climate change challenges. Furthermore, building networks, enhancing communication, sharing information, and leveraging various levels of engagement were also discussed as effective strategies contributing to more resilient solutions.

### Strengths and Limitations

One key gap identified by the scoping review was the lack of empirical investigation of health systems responses to climate change impacts and the role of community action in these efforts. One reason may be that empirical studies of health systems responses to climate-related events, such as natural disasters, do not explicitly include a community-level focus [43]. Accordingly, such studies would not have been identified by our search strategy. Another factor may be how health systems have historically responded to climate change related health vulnerabilities. Increasingly, health policy and systems researchers are encouraged to give the climate change and health nexus greater attention [44]. There has been a trend towards greater attention to this nexus, as, for example, in the recently funded EU-CARIFORUM project on Strengthening Climate Resilient Health Systems in the Caribbean [45].

A limitation of this study is that some relevant articles addressing issues within the scope of the study may have been missed if their title and/or abstract did not include health. Thus, future research should extend the search strategy to include articles that do not include health in their title or abstract but that investigate community-based responses to the wider determinants of health affected by climate change in the anglophone Caribbean.

### Conclusion

As the first scoping study to investigate the current research dedicated to the nexus of climate change, community action, and health in the anglophone Caribbean, this study reveals a diverse set of studies that can inform the field of community action of unique approaches to addressing climate change and health challenges.
