# Supplementary material for: Climate Change, Community Action, and Health in the Anglophone Caribbean: A Scoping Review
Source: Public Health Rev. 2024 Jan 12;44:1605843. doi: 10.3389/phrs.2023.1605843 (PMC10811550; doi:10.3389/phrs.2023.1605843)
Supplement: Supplementary file 1 [file DataSheet1.docx]

Appendix 1: Supplemental File

Title: Search strategies for all databases

**Belmont - Climate change**

| Ovid MEDLINE(R) ALL 1946 to July 12, 2021 (searched 13 July 2021) | 959 |
| --- | --- |
| APA PsycInfo 1806 to July Week 1 2021, Ovid (searched 13 July 2021) | 354 |
| VHL Regional Portal, Virtual Health Library (https://bvsalud.org/en/) (searched 13 July 2021) | 414 |
| Sociological Abstract 1952-Current, ProQuest (searched 13 July 2021) | 658 |
| Scopus, Elsevier (searched 13 July 2021) | 1401 |
| Google Scholar (searched 14 July 2021) | 42 |
|  |  |
| Total before duplicates removed | **3828** |

|  | **Ovid MEDLINE(R) ALL 1946 to July 12, 2021** (searched 13 July 2021) |  |
| --- | --- | --- |
| 1 | Climate Change/ | 19525 |
| 2 | Global Warming/ | 3616 |
| 3 | Sea Level Rise/ | 61 |
| 4 | Greenhouse Effect/ | 5833 |
| 5 | (climat* adj3 (change* or impact* or implication* or effect* or event* or hazard* or threat* or disrupt* or resilience or prepar* or adapt*)).ti,ab,kf. | 53206 |
| 6 | (global warming or greenhouse effect* or green house effect* or sea level rise).ti,ab,kf. | 10801 |
| 7 | or/1-6 [Climate change] | 67927 |
| 8 | Community Participation/ | 17714 |
| 9 | Stakeholder Participation/ | 1601 |
| 10 | Community Networks/ | 7082 |
| 11 | Community-Based Participatory Research/ | 4856 |
| 12 | Citizen Science/ | 184 |
| 13 | Organizations, Nonprofit/ | 3373 |
| 14 | Voluntary Health Agencies/ | 4105 |
| 15 | Faith-Based Organizations/ | 119 |
| 16 | ((community or stakeholder* or local or voluntary or volunteer* or grassroot* or grass root* or activist* or citizen* or civic or civil) adj2 (group* or organisation* or organization*)).ti,ab,kf. | 25682 |
| 17 | ((community or stakeholder* or local or voluntary or volunteer* or grassroot* or grass root* or public or collective or citizen* or civic or civil) adj (participation or involvement or action* or strateg* or respons* or movement* or engagement)).ti,ab,kf. | 31872 |
| 18 | (social movement* or activism or action research or participatory research).ti,ab,kf. | 11896 |
| 19 | or/8-18 [Community action] | 95320 |
| 20 | caribbean region/ or "antigua and barbuda"/ or bahamas/ or barbados/ or belize/ or bermuda/ or british virgin islands/ or dominica/ or grenada/ or guyana/ or jamaica/ or west indies/ or "saint kitts and nevis"/ or saint lucia/ or "saint vincent and the grenadines"/ or "trinidad and tobago"/ or united states virgin islands/ | 16518 |
| 21 | (caribbean* or anguilla or anguillian* or antigua* or barbuda* or bahamas or bahamian* or barbados or barbadian* or belize* or british honduras or bermuda or bermudian* or virgin island* or dominica* or grenada or grenadian* or guyana* or guayana* or guiana or guyanese or jamaica* or west indies or west indian* or cayman islands or caymanian* or montserrat* or turks or caicos or saint kitts or kittian* or st kitts or nevis or nevisian* or saint lucia* or st lucia* or saint vincent or st vincent or vincentian* or grenadine* or greanadine* or trinidad* or tobago* or small island developing state* or sids).ti,ab,kf. | 41072 |
| 22 | or/20-21 [Caribbean] | 44192 |
| 23 | 7 and 22 [Climate change + Caribbean] | 533 |
| 24 | 19 and 22 [Community action + Caribbean] | 469 |
| 25 | 23 or 24 | 990 |
| 26 | english.lg. | 28060732 |
| 27 | 25 and 26 | 959 |

|  | **APA PsycInfo 1806 to July Week 1 2021, Ovid** (searched 13 July 2021) |  |
| --- | --- | --- |
| 1 | Climate Change/ | 2375 |
| 2 | Global Warming/ | 408 |
| 3 | (climat* adj3 (change* or impact* or implication* or effect* or event* or hazard* or threat* or disrupt* or resilience or prepar* or adapt*)).id,ti,ab. | 5905 |
| 4 | (global warming or greenhouse effect* or green house effect* or sea level rise).id,ti,ab. | 813 |
| 5 | or/1-4 | 6562 |
| 6 | Community Involvement/ | 5436 |
| 7 | Action Research/ | 3211 |
| 8 | Nonprofit Organizations/ | 2699 |
| 9 | Faith Based Organizations/ | 352 |
| 10 | Social Movements/ | 4778 |
| 11 | Activism/ | 4773 |
| 12 | ((community or stakeholder* or local or voluntary or volunteer* or grassroot* or grass root* or citizen* or civic or civil) adj2 (group* or organisation* or organization*)).id,ti,ab. | 19777 |
| 13 | ((community or stakeholder* or local or voluntary or volunteer* or grassroot* or grass root* or public or collective or citizen* or civic or civil) adj (participation or involvement or action* or strateg* or respons* or movement* or engagement)).id,ti,ab. | 22372 |
| 14 | (social movement* or activism or action research or participatory research).id,ti,ab. | 22155 |
| 15 | or/6-14 | 68568 |
| 16 | (caribbean* or anguilla or anguillian* or antigua* or barbuda* or bahamas or bahamian* or barbados or barbadian* or belize* or british honduras or bermuda or bermudian* or virgin island* or dominica* or grenada or grenadian* or guyana* or guayana* or guiana or guyanese or jamaica* or west indies or west indian* or cayman islands or caymanian* or montserrat* or turks or caicos or saint kitts or kittian* or st kitts or nevis or nevisian* or saint lucia* or st lucia* or saint vincent or st vincent or vincentian* or grenadine* or greanadine* or trinidad* or tobago* or small island developing state* or sids).id,ti,ab. | 9809 |
| 17 | 5 and 16 | 25 |
| 18 | 15 and 16 | 338 |
| 19 | 17 or 18 | 360 |
| 20 | english.lg. | 4634995 |
| 21 | 19 and 20 | 354 |

| **VHL Regional Portal, Virtual Health Library** (https://bvsalud.org/en/) (searched 13 July 2021) |
| --- |
| Including: LILACS database (Latin American and Caribbean Health Sciences Literature) and MedCarib database. Advanced search - Title, abstract, subject - including all databases except MEDLINE - Limited to English language |
| ("climate change" OR "climate changes" OR "climate effect" OR "climate effects" OR "climate threat" OR "climate threats" OR "climate hazard" OR "climate hazards" OR "climate resilience" OR "global warming" OR "greenhouse effect" OR "greenhouse effects" OR "green house effect" OR "green house effects" OR "sea level rise" OR "community group" OR "community groups" OR "community organization" OR "community organizations" OR "community organisation" OR "community organisations" OR "community participation" OR "community involvement" OR "community action" OR "community engagement" OR "community response" OR "community movement" OR "community movements" OR "stakeholder participation" OR "stakeholder involvement" OR "stakeholder engagement" OR "stakeholder response" OR "public participation" OR "public involvement" OR "public engagement" OR grassroot OR "grass root" OR grassroots OR "grass roots" OR "social movement" OR "social movements" OR activism OR "action research" OR "participatory research" OR "citizen group" OR "citizen groups" OR "citizen organization" OR "citizen organizations" OR "citizen organisation" OR "citizen organisations" OR "citizen participation" OR "citizen involvement" OR "citizen action" OR "citizen engagement" OR "citizen response" OR "citizen movement" OR "citizen movements" OR "civil group" OR "civil groups" OR "civil organization" OR "civil organizations" OR "civil organisation" OR "civil organisations" OR "civil participation" OR "civil involvement" OR "civil action" OR "civil engagement" OR "civil response" OR "civil movement" OR "civil movements" OR "civic group" OR "civic groups" OR "civic organization" OR "civic organizations" OR "civic organisation" OR "civic organisations" OR "civic participation" OR "civic involvement" OR "civic action" OR "civic engagement" OR "civic response" OR "civic movement" OR "civic movements") AND (caribbean* OR anguilla OR anguillian* OR antigua* OR barbuda* OR bahamas OR bahamian* OR barbados OR barbadian* OR belize* OR "british honduras" OR bermuda OR bermudian* OR "virgin island" OR "virgin islands" OR dominica* OR grenada OR grenadian* OR guyana* OR guayana* OR guiana OR guyanese OR jamaica* OR "west indies" OR "west indian" OR "west indians" OR "cayman islands" OR caymanian* OR montserrat* OR turks OR caicos OR "saint kitts" OR kittian* OR "st kitts" OR nevis OR nevisian* OR "saint lucia" OR "saint lucians" OR "st lucia" OR "st lucians" OR "saint vincent" OR "st vincent" OR vincentian* OR grenadine* OR greanadine* OR trinidad* OR tobago* OR "small island developing state" OR "small island developing states" OR sids OR "coastal community" OR "coastal communities") AND ( db:("LILACS" OR "MedCarib" OR "PAHO" OR "PAHOIRIS" OR "DESASTRES" OR "WHOLIS" OR "CidSaude" OR "CUMED" OR "MULTIMEDIA" OR "LIS" OR "SES-SP" OR "BDENF" OR "RHS" OR "BINACIS" OR "HISA" OR "IBECS" OR "LIPECS" OR "PIE" OR "PREPRINT-MEDRXIV") AND la:("en")) |

| **Sociological Abstract 1952-Current, ProQuest** (searched 13 July 2021) |
| --- |
| Limited to English language |
| MAINSUBJECT,TI,AB( "climate change" OR "climate changes" OR "climate effect" OR "climate effects" OR "climate threat" OR "climate threats" OR "climate hazard" OR "climate hazards" OR "climate resilience" OR "global warming" OR "greenhouse effect" OR "greenhouse effects" OR "green house effect" OR "green house effects" OR "sea level rise" OR "community group" OR "community groups" OR "community organization" OR "community organizations" OR "community organisation" OR "community organisations" OR "community participation" OR "community involvement" OR "community action" OR "community engagement" OR "community response" OR "community movement" OR "community movements" OR "stakeholder participation" OR "stakeholder involvement" OR "stakeholder engagement" OR "stakeholder response" OR "public participation" OR "public involvement" OR "public engagement" OR grassroot OR "grass root" OR grassroots OR "grass roots" OR "social movement" OR "social movements" OR activism OR "action research" OR "participatory research" OR "citizen group" OR "citizen groups" OR "citizen organization" OR "citizen organizations" OR "citizen organisation" OR "citizen organisations" OR "citizen participation" OR "citizen involvement" OR "citizen action" OR "citizen engagement" OR "citizen response" OR "citizen movement" OR "citizen movements" OR "civil group" OR "civil groups" OR "civil organization" OR "civil organizations" OR "civil organisation" OR "civil organisations" OR "civil participation" OR "civil involvement" OR "civil action" OR "civil engagement" OR "civil response" OR "civil movement" OR "civil movements" OR "civic group" OR "civic groups" OR "civic organization" OR "civic organizations" OR "civic organisation" OR "civic organisations" OR "civic participation" OR "civic involvement" OR "civic action" OR "civic engagement" OR "civic response" OR "civic movement" OR "civic movements") AND MAINSUBJECT,TI,AB(caribbean* OR anguilla OR anguillian* OR antigua* OR barbuda* OR bahamas OR bahamian* OR barbados OR barbadian* OR belize* OR "british honduras" OR bermuda OR bermudian* OR "virgin island" OR "virgin islands" OR dominica* OR grenada OR grenadian* OR guyana* OR guayana* OR guiana OR guyanese OR jamaica* OR "west indies" OR "west indian" OR "west indians" OR "cayman islands" OR caymanian* OR montserrat* OR turks OR caicos OR "saint kitts" OR kittian* OR "st kitts" OR nevis OR nevisian* OR "saint lucia" OR "saint lucians" OR "st lucia" OR "st lucians" OR "saint vincent" OR "st vincent" OR vincentian* OR grenadine* OR greanadine* OR trinidad* OR tobago* OR "small island developing state" OR "small island developing states" OR sids) |

| **Scopus, Elsevier** (searched 13 July 2021) |
| --- |
| Excluded MEDLINE records - Limited to English language |
| ( ( TITLE-ABS-KEY ( "climate change" OR "climate changes" OR "climate effect" OR "climate effects" OR "climate threat" OR "climate threats" OR "climate hazard" OR "climate hazards" OR "climate resilience" OR "global warming" OR "greenhouse effect" OR "greenhouse effects" OR "green house effect" OR "green house effects" OR "sea level rise" ) ) AND ( TITLE-ABS-KEY ( health* OR resilienc* ) ) AND ( TITLE-ABS-KEY ( caribbean* OR anguilla OR anguillian* OR antigua* OR barbuda* OR bahamas OR bahamian* OR barbados OR barbadian* OR belize* OR "british honduras" OR bermuda OR bermudian* OR "virgin island" OR "virgin islands" OR dominica* OR grenada OR grenadian* OR guyana* OR guayana* OR guiana OR guyanese OR jamaica* OR "west indies" OR "west indian" OR "west indians" OR "cayman islands" OR caymanian* OR montserrat* OR turks OR caicos OR "saint kitts" OR kittian* OR "st kitts" OR nevis OR nevisian* OR "saint lucia" OR "saint lucians" OR "st lucia" OR "st lucians" OR "saint vincent" OR "st vincent" OR vincentian* OR grenadine* OR greanadine* OR trinidad* OR tobago* OR "small island developing state" OR "small island developing states" OR sids ) ) AND NOT INDEX ( medline ) ) OR ( ( TITLE-ABS-KEY ( "community group" OR "community groups" OR "community organization" OR "community organizations" OR "community organisation" OR "community organisations" OR "community participation" OR "community involvement" OR "community action" OR "community engagement" OR "community response" OR "community movement" OR "community movements" OR "stakeholder participation" OR "stakeholder involvement" OR "stakeholder engagement" OR "stakeholder response" OR "public participation" OR "public involvement" OR "public engagement" OR grassroot OR "grass root" OR grassroots OR "grass roots" OR "social movement" OR "social movements" OR activism OR "action research" OR "participatory research" OR "citizen group" OR "citizen groups" OR "citizen organization" OR "citizen organizations" OR "citizen organisation" OR "citizen organisations" OR "citizen participation" OR "citizen involvement" OR "citizen action" OR "citizen engagement" OR "citizen response" OR "citizen movement" OR "citizen movements" OR "civil group" OR "civil groups" OR "civil organization" OR "civil organizations" OR "civil organisation" OR "civil organisations" OR "civil participation" OR "civil involvement" OR "civil action" OR "civil engagement" OR "civil response" OR "civil movement" OR "civil movements" OR "civic group" OR "civic groups" OR "civic organization" OR "civic organizations" OR "civic organisation" OR "civic organisations" OR "civic participation" OR "civic involvement" OR "civic action" OR "civic engagement" OR "civic response" OR "civic movement" OR "civic movements" ) ) AND ( TITLE-ABS-KEY ( caribbean* OR anguilla OR anguillian* OR antigua* OR barbuda* OR bahamas OR bahamian* OR barbados OR barbadian* OR belize* OR "british honduras" OR bermuda OR bermudian* OR "virgin island" OR "virgin islands" OR dominica* OR grenada OR grenadian* OR guyana* OR guayana* OR guiana OR guyanese OR jamaica* OR "west indies" OR "west indian" OR "west indians" OR "cayman islands" OR caymanian* OR montserrat* OR turks OR caicos OR "saint kitts" OR kittian* OR "st kitts" OR nevis OR nevisian* OR "saint lucia" OR "saint lucians" OR "st lucia" OR "st lucians" OR "saint vincent" OR "st vincent" OR vincentian* OR grenadine* OR greanadine* OR trinidad* OR tobago* OR "small island developing state" OR "small island developing states" OR sids ) ) AND NOT INDEX ( medline ) ) AND ( LIMIT-TO ( LANGUAGE , "English" ) ) |

|  | **Google Scholar** (searched 14 July 2021) |
| --- | --- |
|  | allintitle: (community OR stakeholder OR grassroot OR grassroots OR "grass root" OR "grass roots" OR citizen OR civil OR civic) (involvement OR participation OR action OR engagement) (caribbean) |
